# The Prospective Associations of Egg Consumption with the Risk of Total Cerebrovascular Disease Morbidity among Chinese Adults

**DOI:** 10.3390/nu15081808

**Published:** 2023-04-07

**Authors:** Chi Pan, Xiaohui Sun, Jiahui Song, Canqing Yu, Yu Guo, Shaojie Wang, Ruqin Gao, Feng Ning, Zengchang Pang, Zhengming Chen, Liming Li

**Affiliations:** 1 School of Public Health, Qingdao University, Qingdao 266071, China; p1085133@163.com (C.P.);; 2 Qingdao Municipality Center for Disease Control and Prevention, Qingdao 266033, China; 3 Qingdao Institute of Preventive Medicine, Qingdao 266033, China; 4 Department of Epidemiology and Biostatistics, School of Public Health, Peking University Health Science Center, Beijing 100191, China; 5 Chinese Academy of Medical Sciences, Beijing 100730, China; 6 Clinical Trial Service Unit and Epidemiological Studies Unit (CTSU), Nuffield Department of Population Health, University of Oxford, Oxford OX3 7LF, UK

**Keywords:** Chinese, prospective study, egg, cerebrovascular disease

## Abstract

Background: Studies investigating the relationship between egg consumption and the risk of cerebrovascular disease (CED) have yielded inconsistent results. This study evaluated the association between egg consumption and the risk of CED among Chinese adults. Methods: Data were obtained from China Kadoorie Biobank, Qingdao. A computerised questionnaire was used to collect information regarding egg consumption frequency. CED events were tracked through linkage with the Disease Surveillance Point System and the new national health insurance databases. Cox proportional hazards regression analyses were used to evaluate associations between egg consumption and CED risk controlling for potential confounders. Results: After a median follow-up of 9.2 years, 865 and 1083 CED events among men and women, respectively, were documented. More than 50% of participants consumed eggs daily with an average age of 52.0 (10.4) years at baseline. No association between egg consumption and CED were identified in the whole cohort and women. However, a 28% lower risk of CED was observed in those who consumed eggs at a higher frequency (HR = 0.72, 95% CI: 0.55–0.95) and a significant trend for the association (*p* for trend = 0.012) in a multivariable model in men. Conclusion: Higher frequency of egg consumption was associated with a lower risk of total CED events among men but not women in Chinese adults. The beneficial effect on women warrants further investigations.

## 1. Introduction

Cerebrovascular disease (CED) is an umbrella term for a range of disorders that affect the blood vessels that supply blood to the brain. The two main types of CED are haemorrhagic stroke and ischaemic stroke. The Global Burden of Disease 2019 study estimated that all-age disability-adjusted life-years (DALY) due to CED increased by 32.4% and that all-age death counts increased by 43.0% between 1990 and 2019, despite the age-standardised DALY and death rates decreasing during the same period [1,2]. In China, the crude death rate from CED has been increasing rapidly, and as of 2019, CED is the third leading cause of death (149.6 per 100,000). The crude death rate caused by CED was higher in men than in women, and in rural areas than in urban areas [3]. Additionally, it has been reported that the age-specific increase in stroke prevalence was particularly significant among individuals aged older than 60 years [4]. The rising burden of CED has led to a rapid expansion of healthcare costs for Chinese residents.

China has been transitioning from traditional grain diets to animal and plant-based diets over the last three decades [5], resulting in a transformation of macronutrient intake in Chinese diets. This change has disrupted traditional dietary patterns and has been accompanied by a rapid increase in the risk of noncommunicable diseases caused by both undernutrition and overnutrition, such as obesity, hypertension, diabetes and CED [6,7]. Various dietary patterns and functional foods have been demonstrated to improve health. Eggs are considered a healthy food because of the variety of essential nutrients and bioactive components they contain (linoleic acid, vitamin B and carotenoids etc.) which play an important role in anti-inflammatory and lipid-lowering processes [8,9]. While the high amount of cholesterol in egg yolk has led to controversy regarding whether eggs have a protective effect against cardiovascular disease (CVD), since the association between dietary cholesterol intake and dyslipidemia has been consistently inconclusive. For example, in a randomized controlled trial (RCT), cholesterol intake from eggs, with carbohydrate restriction, reduced plasma total cholesterol (TC) concentrations and increased high-density lipoprotein concentrations (HDL-C) [10]. However, another RCT reported that consuming one egg per day did not change the plasma TC or HDL-C levels [11].

Previous studies have provided limited epidemiological evidence on the association between egg consumption and total CED risk and have produced inconsistent results. Mazidi et al. and Qin et al. reported an inverse association between egg consumption and stroke [12,13], whereas Tong et al. and Al-Ramady et al. observed a positive association [14,15], and other studies have reported that no statistical significance was observed [16,17,18]. Heterogeneous results were also obtained across the meta-analysis based on different studies and populations. In a recent meta-analysis of prospective cohort studies, the consumption of at least one egg per day had no significant association with stroke compared with the consumption of less than one egg per month, either in American or in European populations, but had an inverse association in the Asian population [19]. However, another meta-analysis indicated that individuals who consume generally one egg per day had a 12% lower risk of stroke than those who consumed less than two per week [20].

The Chinese Nutrition Society recommends eggs as an affordable and healthy food to ensure a certain amount of intake of every day [21]. However, the China National Nutrition Surveys reported that egg intake has not increased significantly in the past decades and is far below the 300–500 g of eggs per person per week recommended by the 2022 Dietary Guidelines [5]. Thus, a full understanding of egg consumption and its influence on the risk of CED is imperative to Chinese public health. The present study builds on data from the China Kadoorie Biobank (CKB) for a long-term follow-up. We aimed to comprehensively examine the evidence on the association between egg consumption and total CED morbidity adjusting for updated lifestyle and potential dietary confounders.

## 2. Materials and Methods

### 2.1. Study Population

Data were obtained from the CKB in Qingdao. Details about the questionnaire and study procedures have been described elsewhere [22,23]. In brief, a total of 35,508 urban residents aged 30–79 years completed the baseline survey of the CKB study between 2004 and 2008. Respondents who self-reported a history of stroke (*n* = 238) were excluded. A total of 35,270 participants (15,504 men and 19,766 women) were included for analysis in the present study.

Ethics approval was obtained from the Ethics Review Committee of the Chinese Center for Disease Control and Prevention, the Oxford Tropical Research Ethics Committee at the University of Oxford, and the Shandong Provincial and Qingdao Centers for Disease Control and Prevention in China. Informed written consent was obtained from all participants.

### 2.2. Data Collection

In the baseline survey, trained investigators were provided with a self-administered, computerized questionnaire to collect information on sociodemographic characteristics, lifestyle factors, diet, personal and family medical history, physical activities and mental situation etc. Information on diet included the frequency of consumption of foods from the 12 major food groups (rice, wheat, other staple foods, red meat, poultry, fish, eggs, dairy products, fresh fruit, fresh vegetables, soybeans, and preserved vegetables) over the past 12 months, participants were asked, “During the past 12 months, how often did you eat the following foods?” Possible responses were classified into five levels: daily, 4–6 days per week, 1–3 days per week, monthly, and never/rarely. To assess the repeatability of the response, the questionnaire was administered to a subsample of 926 participants within a year (average interval 5.4 months). The frequency of almost all food groups’ consumption were highly reproducible and valid, with the exception of fresh vegetables. As syndicated by an age-adjusted and sex-adjusted Spearman correlation coefficient of 0.58 for egg consumption [24]. Calibrated instruments were provided to measure body height and weight. Total physical activity levels were quantified as the metabolic equivalent of energy task hours per day (MET-h/day) [25]. Body mass index (BMI) was calculated as body weight divided by the square of height (kg/m^2^). Diabetes at baseline included a self-reported history of physician-diagnosed diabetes, current treatment, and screen-detected diabetes, where screen-detected diabetes was defined as a blood glucose level ≥7.0 mmol/L and a fasting time >8 h; a blood glucose level ≥11.1 mmol/L and a fasting time <8 h, or a fasting blood glucose level ≥7.0 mmol/L. Family history of stroke was considered if any parents, siblings, or children (first-degree relative) had received a diagnosis of stroke.

### 2.3. Outcome Variables

Information on incident CED was tracked through linkage with the Disease Surveillance Point System (DSPs) and the new national health insurance databases. Fatal and nonfatal CED events were coded by study clinicians (blinded to baseline information) in accordance with the International Classification of Diseases, 10th Revision. The primary outcome was total CED events (International Classification of Diseases, 10th Revision codes I60–I69), including subarachnoid stroke, haemorrhagic stroke, ischaemic stroke, unspecified stroke and other cerebrovascular disease.

### 2.4. Statistical Methods

All analyses were performed separately by egg consumption levels. Continuous variables with a normal distribution are expressed as means and standard deviations. Categorical variables are expressed as percentages. The distributions of the variables were compared using one-way ANOVA statistical analyses for continuous variables and the chi-square test for categorical variables.

Person-years were calculated from the date of participation until the occurrence of a CED event, loss to follow-up, or 31 December 2015, whichever occurred first. Hazard ratios (HRs) and 95% confidence intervals (CIs) for the associations between egg consumption and the onset of CED were estimated using Cox proportional hazards models. Potential confounding factors were adjusted through the following steps. Model 1 adjusted for age at recruitment date and gender. Model 2 additionally included education level, marital status, household income per year, alcohol consumption status, smoking status, family history of stroke, diabetes at baseline and daily food consumption of rice, wheat, other staple foods, red meat, poultry, fish, dairy products, fresh fruit, soybean products and preserved vegetables. Model 3 additionally included MET and BMI. Given the potential instability of fresh vegetable consumption, we did not include it as a covariable in the maladjusted model.

To investigate the effect of the heterogeneity of baseline characteristics on the association between egg consumption and CED, subgroup analyses based on age (<50 or ≥50 years), education level (below high school or high school and above), marital status (married or unmarried), household income per year (<10,000, 10,000–34,999 and ≥35,000 Chinese Yuan, CNY), alcohol consumption status (currently drinking or not), smoking status (currently smoking or not), family history of stroke (yes or no), diabetes at baseline (yes or no), MET (<18 or ≥18 h/day) and BMI (<24, 24–28 and ≥28 kg/m^2^) were performed. The likelihood ratio test was used to assess the significance of the interaction between the stratification variables and egg consumption. Sensitivity analyses were further adjusted for the ntithrombotic, antihypertensive and lipid-lowering therapy, excluding participants who incident CED within the first 1 year of follow up, as well as excluding participants who incident CED within the first 2 years of follow up. All statistical analyses were performed with SPSS software version 25.0 (SPSS, IBM Corp, Armonk, NY, USA). All graphs were plotted by R statistical software 4.1.1 (R Core Team, Vienna, Austria). A two-sided *p* < 0.05 was defined as statistically significant.

## 3. Results

The distributions of basic characteristics by frequency of egg consumption are summarised in Table 1 and Supplementary Table S1. Among the 35,270 participants, 44% were men. The mean age of men and women was 51.3 (10.4) and 52.5 (10.4) years, respectively. Of the 35,270 participants, 7884 (50.9%) men and 10,577 (53.5%) women consumed eggs 7 days/week, and 994 (6.4%) men and 1199 (6.1%) women reported consuming eggs <1 day/week, respectively. The mean MET and BMI of the overall cohort were 18.1 (11.4) h/day and 25.7 (3.5) kg/m^2^, respectively. Compared with participants who consumed eggs infrequently (<1 day/week), those who consumed eggs at higher levels (7 days/week) were more likely to be older, women, had a higher socioeconomic status, married, noncurrent smoker, had a family history of stroke, had diabetes at baseline, consumed rice, wheat, other staple foods, red meat, dairy products, fresh fruit, fresh vegetables, soybean products, preserved vegetables more frequently, and had fewer MET hours per day (*p* < 0.001 for all comparisons).

The results of the Cox proportional hazards regression analyses are summarised in Table 2. After a median follow-up of 9.2 years, 865 and 1083 CED events among men and women, respectively, were documented, with an incidence of 6.5 per 1000 person-years both in men and women (Supplementary Table S2). A significant trend of lower risk of CED with increasing egg intake was observed, with HR of 0.81 (0.67–0.98) for the highest (7 days/week) and 0.91 (0.72–1.15) for moderate (1–3 days/week) vs. lowest (<1 day/week) egg consumption in model 1 of total participants (*p* for trend = 0.004), however, this association became nonsignificant after the inclusion of additional confounding factors in the whole cohort. Individuals with less frequent egg consumption, the multiadjusted HRs (95% CIs) for consumption of 1–3 days/week, 4–6 days/week and 7 days/week were 0.83 (0.63–1.11), 0.97 (0.70–1.35), and 0.72 (0.55–0.95) in men, respectively (*p* for trend = 0.012). No significant differences were observed in the association between egg consumption and CED risk among women. The association did not change substantially when waist diameter or the waist-to-hip ratio was replaced with BMI in the multivariate model.

In the sensitivity analyses, after excluding the events that occurred in the first 2 years of follow-up and metabolic disease treatment, the significant inverse association between egg consumption and CED risk remained stable. We further included the ntithrombotic, antihypertensive and lipid-lowering therapy or excluding the participants who had an incidence of CED within the first 1 year of follow-up, the HR in men who consumed eggs 7 days/week was 0.73 (0.55, 0.96) and 0.70 (0.53, 0.93), respectively. In addition, after excluding the CED events that occurred within the first 2 years of follow-up, the association become moderate. The multiadjusted HR for CED of men with the highest level of egg consumption was 0.74 (0.55, 1.00) (Supplementary Table S3).

**Figure 1:**
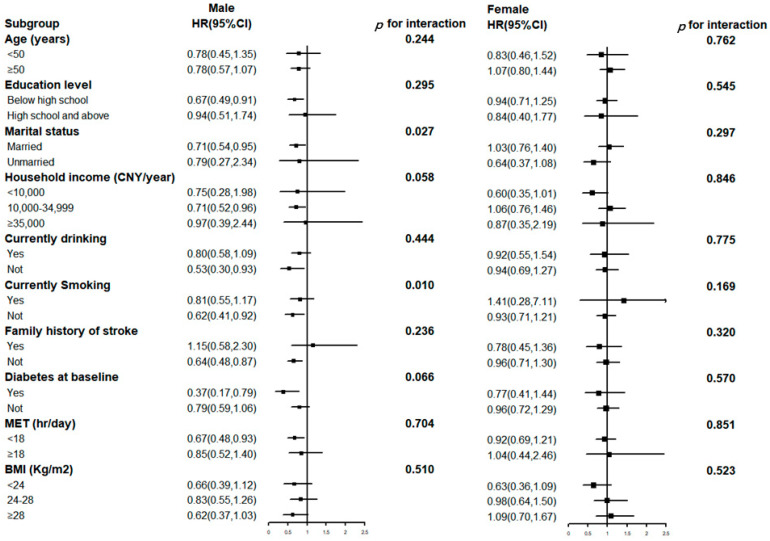
Subgroup analyses of the association between egg consumption and cerebrovascular disease (CED) according to baseline characteristics. Multivariate models were adjusted for age, gender, education level, marital status, household income per-year, alcohol consumption status, smoking status, daily food consumption of rice, wheat, other staple foods, red meat, poultry, fish, dairy products, fresh fruit, soybean products, preserved vegetables, MET, BMI, family history of stroke and prevalent diabetes at baseline. Black squares indicate point estimates, horizontal lines indicate the 95% CIs. The tests for interaction were performed using likelihood ratio tests. *p* for interaction: comparing models with and without interaction terms between the strata variable and egg consumption. HR: hazard ratio; CI: confidence interval; CNY: Chinese Yuan; MET: Metabolic Equivalent of Energy; BMI: Body Mass Index.

Additional subgroup analyses based on baseline factors were presented in Figure 1. The same relationships were observed between subgroups stratified by age, education level, household income per year, alcohol consumption status, family history of stroke, diabetes at baseline, MET, and BMI (*p* for interaction > 0.05). A significant interaction was observed among subgroups stratified by marital status (*p* for interaction = 0.027) and smoking status (*p* for interaction = 0.010) in men. We confirmed that the protective effect of egg consumption against CED was stronger in participants who were married and not currently smoking. No statistically significant interactions were observed in women.

## 4. Discussion

In this large population-based prospective study, we observed no significant inverse association between self-reported egg consumption frequency and total CED-related morbidity after adjusting for potential risk factors in the whole cohort. However, among men, those who consumed eggs frequently had a 28% lower risk of CED than did those who consumed eggs less than once per week.

Our findings are inconsistent with those of other studies. In a US representative sample, discovered that when both men and women were included in a multivariate Cox regression analysis, consuming more than six eggs per week had no effect on the risk of stroke or coronary artery disease [26]. It examined the strong associations of egg consumption with cardiovascular disease (CVD) and all-cause mortality by using data from the Guangzhou Biobank Cohort Study of China. After a median follow-up of 9.8 years, no significant associations were observed between egg consumption and mortality from CVD, ischaemic heart disease, or stroke [27]. In a multiethnic population-based cohort study that investigated stroke incidence, risk factors, and prognosis, Goldberg et al. evaluated the associations of egg consumption with carotid atherosclerosis phenotypes, and the risk of clinical vascular outcomes. They observed an inverse correlation between low-to-moderate egg consumption and carotid intima-media thickness and plaque presence, thickness, and area; however, no significant negative trend was observed for combined vascular events, stroke, myocardial infarction, and vascular death with increased egg consumption frequency [28]. Similarly, Bernstein et al. [29] reported no association between egg consumption and stroke risk in an analysis that included men and women.

Sex-specific differences in the relationship between egg consumption and CED events have rarely been noted in the literature, especially in Asian populations. A prospective study using data from the Third National Health and Nutrition Examination Survey (NHANES; 1988–2000) evaluated the relationship between egg consumption and mortality from coronary heart disease and stroke and yielded similar results [30]. After an average follow-up of 8.9 years, they observed a statistically significant decrease in stroke mortality among men (HR = 0.27, 95% CI: 0.10–0.73) but not among women (HR = 1.03, 95% CI: 0.25–4.22) who consumed eggs more frequently after adjustment for potential confounders. The significant inverse association between egg consumption and CED mortality risk in men but not women has been confirmed again in the same cohort (NHANES; 1999–2011) in 2019 [12]. By contrast, prospective cohort studies by Gaziano et al. and Nakamura et al. [31,32] observed no significant relationship between egg consumption and stroke mortality in either men or women. Few dietary variables were controlled for in these two studies, other than alcohol and vegetable consumption, whereas our data allowed for more dietary factors, which may be potential confounders in this relationship, to be considered. Within the CKB study, Qin et al. [13] using a sample of data from 10 different geographical survey sites in China discovered that frequent egg consumption was associated with a 26% lower risk of haemorrhagic stroke and a 10% of ischaemic stroke among men and women. The discrepancy between our results and those of Qin et al. may be because our analysis included a regionally representative sample of urban areas and additional total CED events. Data from the current study, egg consumption was positively associated with higher socioeconomic status, non-smoking, lower physical activity in both men and women. In addition, an apparent positive association between higher egg consumption and the CED in men with interaction between non-smoker and married, but not in women, adjusting for socioeconomic status and other confounders. The mechanisms of interaction responsible for this reduced risk require further study. Additionally, the estrogen level, menopause and potential factors related to metabolism and CED were not available and cannot be evaluated in the current study. The sex difference between egg consumption and CED morbidity warrants further investigation.

Subgroup analysis indicated a strong inverse correlation between egg consumption and incident CED among participants who have been married and not currently smoking. Smoking is a significant risk factor for CED [33]. Findings from the Australian prospective study of cardiovascular risk showed that of 36 subtypes of CVD, 29 event rates were significantly increased in current smokers. Adjusted HRs in current smokers were 2.16 (1.93–2.42) and 2.26 (1.65–3.10) for CED morbidity and mortality, respectively [34]. The underlying mechanisms by which smoking affects CED are numerous and include endothelial dysfunction, prothrombotic effects, inflammation, altered lipid metabolism, decreased supply of myocardial blood and oxygen, and insulin resistance [35]. The synergistic effect of marital status and noncurrent smoking on healthy dietary patterns and reduced risk of CED will be discussed in further research.

Eggs are considered a healthy food because of the variety of essential nutrients they contain, and they are also relatively economic. Generally, eggs contain relatively high amounts of dietary cholesterol (350 mg/100 g), which are regulated by the lipid metabolism and has a non-effect on the body, but increase the risk of CVD of individuals with high cholesterol concentrations. Although the high amount of cholesterol in egg yolks has led to controversy regarding their status as a healthy food and their contribution to the risk of CVD [36,37,38], evidence suggests that nutrients derived from eggs have protective effects against CED. There are several possible mechanisms of protection hypotheses. Egg-derived bioactive proteins, such as ovotransferrin and phosvitin, enact immunomodulatory and anti-inflammatory effects by reducing the gene expression of proinflammatory cytokines [39]. The decrease of the concentration of inflammatory mediators such as interleukin-1β, interferon-γ, tumour necrosis factor-α as well as interleukin-8 prevents the development of atherosclerotic plaques and excessive activation of platelets and the coagulation system [40,41]. In addition, eggs contain a substantial amount of high-quality protein, which tends to increase satiety and reduce variation in plasma glucose and insulin levels [42] The relative stability of insulin levels and blood glucose levels prevents the development of insulin resistance, thereby reducing the risk of the level of chronic inflammation [43] and improving coagulopathy [44] and oxidative stress [45], which in turn reduces the risk of stroke and hypertension [46]. Moreover, egg-derived phospholipids preferentially combine with high-density lipoprotein fractions to increase bioavailability and regulate plasma lipids levels by reducing total cholesterol, low-density lipoprotein levels, and triglycerides and by increasing high-density lipoprotein levels [47,48]. The improved low-density-to-high-density lipoprotein ratio can reduce the risk of atherosclerosis. Furthermore, eggs are rich in omega-3 polyunsaturated fatty acids, and the omega-3 polyunsaturated fatty acids were considered to reduce triglyceride concentrations through enhanced fatty acid oxidation may also explain the preventive effect of egg consumption on stroke [49,50]. Besides, the relationship between changes in gut microbiota composition and function and CVD risk has received widespread interest in recent years. It found that intake of 2 eggs every day for two weeks could significantly improve the flow-mediated dilation response and slow down brachial-ankle pulse wave velocity, and effectively modulated the gut microbiota function on the premise of maintaining the homeostasis of intestinal flora, including downregulation of tryptophanase [51]. Enhanced tryptophan degradation is thought to be associated with inflammation [52]. It is also notable that nutritionists have made great achievements in improving egg quality in recent years. Janmohammadi et al. [53] and Sabet et al. [54] reported that an appropriate raw amaranth grain with enzyme blend or hibiscus sabdariffa leaf extract supplemented in laying hens diet can effectively reduce the cholesterol content of egg yolks. Another study [55] demonstrated an increase in egg weight without changing their chemical composition after ingestion of allium extract as a dietary supplement. Unfortunately, the current study did not include the ingredients of egg and their interaction between other food types and should be confirmed in the further research.

The strengths of the present study include its large sample size, population-based design, relatively long follow-up, and low loss to follow-up. The detailed information collected during the survey enabled us to assess the effects of lifestyle, health status, and dietary factors on the relationship between egg consumption and CED events. In addition, control of outcome variables enabled us to track the major stroke subtypes and other cardiovascular events. The study also has several limitations. First, egg consumption being obtained from a non-validated qualitative food frequency questionnaire restricted us from considering specific dose–response relationships. Additionally, the cooking style may attenuate or strengthen the effect of egg protein and yolk absorption on body metabolism. The missing information will be collected and considered in a further investigation. Second, several potential confounders to CED, such as total cholesterol and triglycerides levels, were not included in the current study. Third, the self-report nature of the food frequency data may have introduced recall bias. Finally, the study individuals including the urban residents, the rural counterparts were not available. Caution should be applied when using the current results to generalize to the whole population.

## 5. Conclusions

High-frequency egg consumption is associated with a significantly lower risk of total CED events among men in urban areas of China. These findings may have significant evidence on the nutritional intake patterns in China. The conclusion will be further validated in a long-term follow-up and generalized population.

## Figures and Tables

**Table 1:** Basic characteristics of study participants by frequency of egg consumption.

Characteristics	<1 Day/Week	1–3 Days/Week	4–6 Days/Week	7 Days/Week	Overall
	(*n* = 2193)	(*n* = 10,658)	(*n* = 3958)	(*n* = 18,461)	(*n* = 35,270)
Age (years)	49.1 (9.4)	49.3 (9.6)	49.5 (9.7)	52.0 (10.4)	50.7 (10.1)
Men (%)	45.3	46.2	43.1	42.7	44.0
High school and above	32.0	35.4	40.4	35.2	35.7
Married (%)	91.3	92.3	94.0	92.4	92.4
Household income (CNY/year)					
<10,000	10.1	7.9	5.3	8.1	7.8
10,000–34,999	75.5	76.5	77.2	74.4	75.4
≥35,000	14.4	15.6	17.5	17.5	16.7
Current Smoking	27.9	26.1	25.8	26.4	26.3
Current drinking (%)	63.1	63.7	63.9	63.6	63.6
Eating daily (%)					
rice	17.0	14.4	12.3	20.5	17.5
wheat	84.9	87.9	85.9	88.7	87.9
other staple foods	3.9	2.4	2.1	4.2	3.4
red meat	58.9	56.1	45.2	68.5	61.6
poultry	2.2	2.3	3.4	1.6	2.0
fish	13.5	10.6	6.4	13.0	11.6
dairy products	27.9	24.6	20.9	43.4	34.2
fresh fruit	48.3	47.3	47.0	60.5	54.2
fresh vegetables	96.9	97.2	97.4	99.0	98.1
soybean products	5.6	4.8	4.7	9.4	7.2
preserved vegetables	32.0	25.2	17.5	35.8	30.3
Family history of stroke (%)	18.7	15.3	11.5	17.2	16.1
Diabetes (%)	3.9	4.1	3.9	8.0	6.1
MET (MET-h/day)	19.4 (11.9)	18.7 (11.6)	19.2 (11.8)	17.5 (11.2)	18.1 (11.4)
BMI (kg/m^2^)	25.7 (3.6)	25.7 (3.5)	25.6 (3.5)	25.7 (3.5)	25.7 (3.5)

Data are presented as means (standard deviation) or percentages. CNY: Chinese Yuan; MET: Metabolic Equivalent of Energy; BMI: Body Mass Index.

**Table 2:** Hazard ratios (HRs) and 95% confidence intervals (CIs) for Cerebrovascular Events According to Egg Consumption.

	Egg Consumption				*p* for Trend
All	<1 Day/Week	1–3 Days/Week	4–6 Days/Week	7 Days/Week	
Overall cohort					
Cases	121	532	186	1109	
PYs	19,070	90,422	31,439	158,115	
Cases/PYs (1/1000)	6.3	5.9	5.9	7.0	
Model 1	1.00	0.91 (0.75, 1.11)	0.91 (0.72, 1.15)	0.81 (0.67, 0.98) *	0.004
Model 2	1.00	0.91 (0.75, 1.11)	0.91 (0.72, 1.15)	0.82 (0.68, 1.00)	0.014
Model 3	1.00	0.91 (0.75, 1.11)	0.92 (0.73, 1.16)	0.83 (0.69, 1.01)	0.027
Men					
Cases	59	237	92	477	
PYs	8745	42,273	13,681	68,411	
Cases/PYs (1/1000)	6.7	5.6	6.7	7.0	
Model 1	1.00	0.82 (0.62, 1.09)	0.92 (0.66, 1.28)	0.71 (0.54, 0.94) *	0.008
Model 2	1.00	0.83 (0.62, 1.11)	0.97 (0.69, 1.35)	0.72 (0.54, 0.94) *	0.009
Model 3	1.00	0.83 (0.63, 1.11)	0.97 (0.70, 1.35)	0.72 (0.55, 0.95) *	0.012
Women					
Cases	62	295	94	632	
PYs	10,325	48,149	17,758	89,704	
Cases/PYs (1/1000)	6.0	6.1	5.3	7.0	
Model 1	1.00	1.00 (0.76, 1.31)	0.90 (0.65, 1.24)	0.90 (0.69, 1.18)	0.161
Model 2	1.00	0.98 (0.74, 1.29)	0.86 (0.62, 1.19)	0.91 (0.70, 1.19)	0.316
Model 3	1.00	0.97 (0.74, 1.28)	0.87 (0.62, 1.20)	0.93 (0.71, 1.21)	0.452

Model 1: age at recruitment date, gender (only in whole cohort); Model 2: additionally included education level, marital status, household income per-year, alcohol consumption status, smoking status, family history of stroke, diabetes at baseline, daily food consumption of rice, wheat, other staple foods, red meat, poultry, fish, dairy products, fresh fruit, soybean products, and preserved vegetables; Model 3: additionally included MET and BMI; MET: Metabolic Equivalent of Energy; BMI: Body Mass Index; PYs: Person-years; * *p* < 0.05.

## Data Availability

Details of how to access China Kadoorie Biobank data and details of the data release schedule are available from www.ckbiobank.org/site/Data+Access (accessed on 15 January 2022).

## Supplementary Materials

The following supporting information can be downloaded at: https://www.mdpi.com/article/10.3390/nu15081808/s1, Table S1: Basic characteristics of study participants by gender and frequency of egg consumption; Table S2: Age and sex-specific incidence rates of cerebrovascular disease; Table S3: Sensitivity analyses of egg consumption and risk of cerebrovascular morbidity among Chinese adult.
