# Supplementary material for: The Prospective Associations of Egg Consumption with the Risk of Total Cerebrovascular Disease Morbidity among Chinese Adults
Source: Nutrients. 2023 Apr 7;15(8):1808. doi: 10.3390/nu15081808 (PMC10142563; doi:10.3390/nu15081808)
Supplement: Supplementary file 1 [file nutrients-15-01808-s001.zip › nutrients-2317207-supplementary.pdf]

Supplementary Table S1. Basic characteristics of study participants by gender and frequency of egg consumption.

Characteristics	Men				Women			
	<1 day/week	1–3 days/week	4–6 days/week	7 days/week	<1 day/week	1–3 days/week	4–6 days/week	7 days/week
	(*n* = 994)	(*n* = 4919)	(*n* = 1707)	(*n* = 7884)	(*n* = 1199)	(*n* = 5739)	(*n* = 2251)	(*n* = 10,577)
Age (years)	48.0 (9.0)	48.1 (9.1)	48.9 (9.3)	51.3 (10.4)	50.1 (9.6)	50.3 (10.0)	49.9 (10.0)	52.5 (10.4)
High school and above (%)	33.8	41.8	46.1	40.8	30.4	29.9	36.0	31.0
Married (%)	94.4	95.7	97.3	96.4	88.8	89.3	91.4	89.4
**Household income (CNY/yr, %)**								
<10,000	5.9	4.2	2.5	5.1	13.6	11.1	7.4	10.3
10,000–34,999	78.8	77.8	76.5	75.6	72.8	75.3	77.8	73.6
≥35,000	15.3	18.0	21.0	19.4	13.6	13.6	14.8	16.1
Current Smoking (%)	64.4	58.4	56.5	58.4	1.6	1.2	0.6	1.0
Current drinking (%)	85.5	85.8	88.6	87.6	44.5	44.7	45.2	45.7
**Eating daily (%)**								
rice	15.5	12.7	10.5	18.8	18.3	15.8	13.6	21.7
wheat	87.1	89.5	87.5	89.9	83.0	86.6	84.7	87.8
staple foods	4.2	2.2	1.8	3.6	3.7	2.5	2.3	4.6
red meat	64.8	63.2	52.4	75.6	54.0	50.1	39.8	63.3
poultry	3.2	2.8	3.5	2.2	1.4	1.8	3.3	1.1
fish	17.7	13.7	8.0	16.8	10.1	7.9	5.2	10.2
dairy products	27.1	22.9	18.7	43.4	28.5	26.1	22.5	43.5
fresh fruit	33.6	32.9	34.3	46.2	60.6	59.7	56.6	71.1
fresh vegetables	96.0	96.7	97.2	98.6	97.6	97.7	97.6	99.3
soybean products	6.1	5.0	5.6	10.1	5.1	4.5	4.1	8.9
preserved vegetables	32.1	27.4	20.6	39.5	31.9	23.4	15.1	33.1
Family history of stroke (%)	17.0	14.8	11.9	16.5	20.2	15.8	11.2	17.7
Diabetes (%)	3.0	3.7	3.2	6.9	4.7	4.4	4.5	8.7
MET (MET-hr/day)	22.3 (12.4)	21.4 (12.1)	21.5 (12.5)	19.8 (12.3)	17.0 (10.8)	16.4 (10.7)	17.5 (10.9)	15.8 (9.8)
BMI (kg/m^2)	25.6 (3.3)	25.5 (3.2)	25.3 (3.2)	25.4 (3.2)	25.9 (3.8)	25.9 (3.7)	25.7 (3.7)	25.9 (3.6)

Data are presented as means (standard deviation) or percentages. CNY: Chinese Yuan; MET: Metabolic Equivalent of Energy; BMI: Body Mass Index.

Supplementary Table S2. Age and sex-specific incidence rates of cerebrovascular disease.

Age(years)	**Men**			**Women**			**Overall cohort**		
	Cases	PYs	Cases/PYs(1/1000)	Cases	PYs	Cases/PYs(1/1000)	Cases	PYs	Cases/PYs(1/1000)
30-<40	28	23,838	1.2	14	24,020	0.5	42	47,858	0.9
40-<50	151	54,050	2.8	135	60,404	2.2	286	114,454	2.5
50-<60	280	36,194	7.7	322	47,772	6.7	602	83,966	7.2
60-<70	254	14,093	18.0	380	25,655	14.8	634	39,748	16.0
≥70	152	4935	30.8	232	8085	28.7	384	13,020	29.5
All	865	133,110	6.5	1,083	165,936	6.5	1,948	299,046	6.5

PYs: Person-years

Supplementary Table S3. Sensitivity analyses of egg consumption and risk of cerebrovascular morbidity among Chinese adult.

	<1 day/week	7 days/week		
		**Men**	**Women**	**Overall cohort**
Model 3 + Further adjusted for ntithrombotic, antihypertensive and lipid-lowering therapy	1.00	0.73 (0.55, 0.96) *	0.94 (0.72, 1.22)	0.84 (0.69, 1.02)
Model 3 + Excluding participants who incident CED within the first 1 year of follow-up	1.00	0.70 (0.53, 0.93) *	0.92 (0.70, 1.20)	0.82 (0.67, 0.99) *
Model 3 + Excluding participants who incident CED within the first 2 years of follow-up	1.00	0.74 (0.55, 1.00)	0.94 (0.70, 1.25)	0.85 (0.69, 1.04)

Model 3: age at recruitment date, gender (only in whole cohort), education level, marital status, household income per-year, alcohol consumption status, smoking status, family history of stroke, diabetes at baseline, daily food consumption of rice, wheat, other staple foods, red meat, poultry, fish, dairy products, fresh fruit, soybean products, and preserved vegetables, MET and BMI. * $p < 0.05$
